# Measurement properties and normative data for the Norwegian SF-36: results from a general population survey

**DOI:** 10.1186/s12955-017-0625-9

**Published:** 2017-03-14

**Authors:** AM. Garratt, K. Stavem

**Affiliations:** 1 Knowledge Centre for the Health Services, Norwegian Institute of Public Health, PO Box 4404, Nydalen, N-0403 Oslo Norway; 2 Institute of Clinical Medicine, University of Oslo, Oslo, Norway; 3 Department of Pulmonary Medicine, Medical Division, Akershus University Hospital, Oslo, Norway; 4 Health Services Research Unit, Akershus University Hospital, Oslo, Norway

## Abstract

**Background:**

The interpretation of the SF-36 in Norwegian populations largely uses normative data from 1996. This study presents data for the general population from 2002–2003 which has been used for comparative purposes but has not been assessed for measurement properties.

**Methods:**

As part of the Norwegian Level of Living Survey 2002–2003, a postal survey was conducted comprising 9,164 members of the general population aged 16 years and over representative for Norway who received the Norwegian SF-36 version 1.2. The SF-36 was assessed against widely applied criteria including data completeness and assumptions relating to the construction and scoring of multi-item scales. Normative data are given for the eight SF-36 scales and the two summary scales (PCS, MCS) for eight age groups and gender.

**Results:**

There were 5,396 (58.9%) respondents. Item levels of missing data ranged from 0.6 to 3.0% with scale scores computable for 97.5 to 99.8% of respondents. All item-total correlations were above 0.4 and were of a similar level with the exceptions of the easiest and most difficult physical function items and two general health items. Cronbach’s alpha exceeded 0.8 for all scales. Under 5% of respondents scored at the floor for five scales. Role-physical had the highest floor effect (14.6%) and together with role-emotional had the highest ceiling effects (66.3-76.8%). With three exceptions for the eight age groups, females had lower scores than males across the eight health scales. The two youngest age groups (<30 years) had the highest scores for physical aspects of health; physical function, role-physical, bodily pain and general health. The age groups 40–49 and 60–69 years had the highest scores for role-emotional and mental health respectively.

**Conclusions:**

This SF-36 data meet necessary criteria for applications of normative data. The data is more recent, has more respondents including older people than the original Norwegian normative data from 1996, and can help the interpretation of SF-36 scores in applications that include clinical and health services research.

## Background

The Short Form 36 (SF-36) Health Survey is the most evaluated health status instrument and the most reported within randomized controlled trials [1, 2]. The instrument has been translated into many languages and the results of these studies are published in peer-reviewed journals [3]. SF-36 Version 1 [4] and the RAND-36 [5] include the same items and continue to be widely used, including in the great majority of Norwegian studies that include this instrument. The SF-36 is available in self- or interview-administered formats and standard (four weeks) and acute (one week) recall periods.

The SF-36 was developed as part of the Medical Outcomes Study (MOS), a key objective of which was to develop more practical tools for monitoring the outcomes of medical care [4, 6, 7]. The instrument includes 36 items or questions that assess functional health and well-being from the perspective of the patient. The items contribute to eight health domains of physical functioning, role limitations due to physical problems, bodily pain, general health, vitality, social functioning, role limitations due to emotional problems and mental health. The eight domains all contribute to physical component summary (PCS) and mental component summary (MCS) scores, with their relative weights based on the results of factor analysis [8]. Short-forms include the SF-12 [9] and SF-8 [10] which give summary scores along with single item scores for each domain in the case of the latter.

Normative data derived from surveys of representative samples of the general population aid the interpretation of the SF-36 scale and summary scores [11]. Normative data has been available following early evaluations of the instrument, for example as part of the International Quality of Life Assessment (IQOLA) Project [3, 12]. Much of this data was collected in the 1990s following forward backward translations and testing for cross-cultural equivalence [3, 13, 14]. These normative data continue to be used [15–17] but more recent data is available for countries that were not included in the IQOLA Project [18–20].

The Norwegian SF-36 version 1.1 was forward backwards translated according to the IQOLA procedures and evaluated in patients with rheumatoid arthritis recruited from a patient register for Oslo [21]. Problems with missing data and suboptimal psychometric characteristics led to slight revisions to five items in version 1.2 [12], the one commonly used in Norway. This version was evaluated in a nationally representative sample of the Norwegian general population in the spring of 1996 and was used to derive the Norwegian norms [12]. The data is over 20 years old and may no longer be representative of the general population due to changes in both the composition of the general population and how individuals respond to such questions.

The present study presents more recent normative data for the Norwegian SF-36 v1.2 [22]. This data has been used to help the interpretation of SF-36 scores in Norwegian studies since 2013 [23–25]. Compared to the original Norwegian norms [12], there are a larger number of respondents including older people, which further contributes to the appropriateness of this new normative data. However, the measurement properties of this normative data have not been reported. Norms are also given for the SF-36 summary scales, which were developed later and hence were not included in the original normative data. The study also presents norms for the two scales that have a different scoring algorithm according to the RAND scoring together with alternative scoring for the summary scales [26–28]. The present study follows the IQOLA project and existing studies that have evaluated the SF-36 in general populations including tests of data quality and internal consistency.

## Methods

### Data collection

**Fig. 1:**
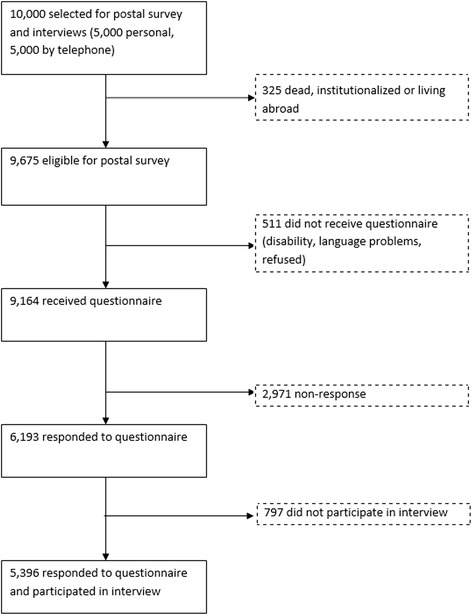
Data Collection

The postal survey comprised 9,164 members of the general population aged 16 years and over that were representative for Norway (Fig. 1). It was conducted as part of the Norwegian “Level of Living Survey 2002” cross sectional study on health undertaken by Statistics Norway and included home and telephone interviews prior to the postal survey [22]. The postal questionnaire included the Norwegian SF-36 version 1.2 mailed in the period 15 November 2002 to 15 May 2003. SF-36 data were available for the 5,396 interview participants only from the Norwegian Social Science Data Services AS (NSD).

### Measurement properties

The analysis followed the measurement criteria evaluated as part of the IQOLA project that included the Norwegian version of the SF-36 [3]. Data completeness was evaluated by considering the percentage of respondents with missing data at the item and scale levels including the percentage of scale scores calculable according to the SF-36 scoring. According to classical test theory and the construction of summated rating scales, item means are expected to be roughly equal but this is seldom the case due to heterogeneity of item content. For the physical functioning scale it was hypothesized that items assessing the least strenuous activities would have the highest mean scores and that the climbing stairs and walking items would have item means ordered as a Guttman scale. For the two role functioning scales it was hypothesized that the items relating to “accomplished less than you would like” would have the lowest item means. For the vitality scale it was hypothesized that vitality items assessing well-being would have lower mean scores than items assessing disability, since the former define higher levels of health. For the mental health scale it was hypothesized that items assessing positive affect would have lower item means than those assessing negative affect. Internal consistency was assessed by item-total correlation and Cronbach’s alpha. Item-total correlations of 0.4 or higher were considered satisfactory and should be approximately equal within each scale [3]. Definite scaling success was defined as an item correlating by two standard errors or more with its scale than with another scale and probable scaling success when the correlation was higher but not by two standard errors [3]. Cronbach’s alpha should be at least 0.70 and 0.90 for group and individual level analyses respectively [3]. Floor and ceiling effects were assessed through the percentage of respondents with the lowest and highest scale scores.

### Normative data

Normative data are presented in the same manner as previous SF-36 studies and are broken down by age and gender [12, 14]. For the PCS and MCS, normative data are given for the standard scoring derived using an uncorrelated (orthogonal) factor solution [8] and scoring based on a correlated (oblique) factor solution [26]. The former is based on data for the general population of the US standardized to have a mean of 50 and standard deviation of 10 [8]. The latter uses weights derived from an oblique factor solution [26] standardized to have a mean of 50 and standard deviation of 10 in the current sample. The RAND scoring of the SF-36 is an alternative scoring for the same questionnaire (here Norwegian version 1.2). It has slightly different scoring for the bodily pain and general health scales. This study gives normative data for these scales alongside the alternative scoring for the PCS and MCS.

IBM SPSS 23 was used for descriptive statistics and to assess the measurement properties.

## Results

### Data collection

Of 9,675 eligible members of the general population, 511 people did not receive a questionnaire because of disability, language difficulties, or they refused. Of the 9,164 who received a questionnaire, SF-36 data were available for the 5,396 (55.8%) respondents who had also participated in the interviews (Fig. 1) and their background characteristics are shown in Table 1 [22].

**Table 1:** Characteristics of respondents (*n* = 5396)

	Number	Percent
Age, years; mean (SD)	46.57 (17.44)	
Age category, years	296	5.49
15–19 yrs		
20–29	729	13.51
30–39	1016	18.83
40–49	1080	20.01
50–59	980	18.16
60–69	675	12.51
70–79	439	8.14
80-	181	3.35
Gender		
Female	2773	51.39
Male	2623	48.61
Marital status^a^		
Divorced/separated	441	8.17
Cohabitant/married	2964	54.93
Single	1679	31.12
Widowed	311	5.76
Education^b^		
Under 10 yrs	839	16.49
10 – 12 yrs	2746	53.97
University (>12 yrs)	1503	29.54

^a^Missing data for one respondent

^b^Missing data for 141 respondents

### Measurement properties

Table 2 shows that the item levels of missing data ranged from 0.6 to 3.0% for the bodily pain item “how much did pain interfere with your normal work” and general health item “I seem to get sick easier than others” respectively. Levels of complete data for the eight scales ranged from 95.4 to 98.6% for general health and social functioning respectively. Following score computation the level of missing data ranged from 0.2 to 2.5% for these two scales. Levels of missing data were slightly higher for the summary scales, which are dependent on complete data for scale scores.

**Table 2:** Descriptive statistics and internal consistency (*n* = 5396)

Scale/item	Percent missing	Percent complete data	Mean	Standard deviation	Percent floor	Percent ceiling	Cronbach’s alpha (scale)/item scale correlation	Percent scaling success
Physical functioning	0.87	95.37	86.44	20.42	0.45	35.60	0.92	97.50
1 Vigorous activites	1.54		2.16	0.76	22.55	38.15	0.55	75
2 Moderate activities	1.26		2.71	0.56	5.61	76.71	0.83	100
3 Lifting or carrying groceries	1.46		2.79	0.50	4.29	83.49	0.76	100
4 Climbing several flights of stairs	1.15		2.71	0.58	6.22	76.90	0.80	100
5 Climbing one flight of stairs	1.61		2.87	0.40	2.41	89.83	0.75	100
6 Bending, kneeling, stooping	1.17		2.69	0.59	6.43	75.51	0.74	100
7 Walk more than a mile	1.35		2.70	0.61	7.95	77.46	0.79	100
8 Walking several blocks	1.70		2.87	0.42	3.15	89.69	0.78	100
9 Walking one block	1.98		2.91	0.34	1.85	92.93	0.70	100
10 Bathing or dressing	0.89		2.91	0.34	1.66	92.89	0.61	100
Role-physical	0.85	97.59	76.64	37.39	14.62	66.32	0.91	100
1 Cut down time spent on work	1.15		1.81	0.40	19.35	80.65	0.78	100
2 Accomplished less than would like	1.02		1.72	0.45	27.61	72.39	0.76	100
3 Limited in kid of work/activities	1.76		1.77	0.42	23.30	76.70	0.80	100
4 Difficulty performing work/activities	0.95		1.77	0.42	22.60	77.40	0.83	100
Bodily pain^a^	0.26	97.70	73.62	25.83	0.63	35.56	0.90	100
1 Intensity of bodily pain	1.98		4.67	1.40	1.91	36.66	0.82	100
2 Extent pain interfered with work	0.57		4.69	1.31	2.05	35.58	0.82	100
General health^a^	2.52	95.89	75.25	21.72	0.23	8.21	0.82	100
1 Rating of general health	0.61		3.68	1.04	3.56	12.40	0.68	100
2 I seem to get sick easier than others	3.04		4.44	1.03	2.01	73.13	0.51	100
3 I seem as healthy as anyone I know	2.19		4.26	1.10	3.58	59.44	0.63	100
4 I expect my health to get worse	2.59		3.74	1.26	4.85	42.69	0.52	100
5 My health is excellent	2.46		3.92	1.31	6.56	39.83	0.76	100
Vitality	1.22	95.79	60.72	20.61	0.73	2.40	0.85	100
1 Full of pep	2.46		3.62	1.31	6.75	6.38	0.64	100
2 Have a lot of energy	2.26		3.55	1.36	8.57	6.12	0.75	100
3 Feel worn out	1.85		4.52	1.15	2.59	16.11	0.68	100
4 Feel tired	1.30		4.45	1.13	2.22	13.31	0.70	100
Social Functioning	0.17	98.85	86.27	21.18	0.84	58.40	0.81	100
1 Extent health problems interfered	0.61		4.51	0.87	1.55	68.73	0.69	100
2 Frequency health problems interfered	0.70		4.40	0.95	2.11	63.55	0.69	100
Role-Emotional	1.58	97.72	84.23	31.67	8.53	76.84	0.84	100
1 Cut down time spent on work	1.41		1.87	0.34	12.93	87.07	0.71	100
2 Accomplished less than would like	1.46		1.80	0.40	20.50	79.50	0.74	100
3 Work not done as carefully as usual	1.65		1.86	0.35	13.96	86.04	0.68	100
Mental Health	1.57	95.53	80.27	15.47	0.13	6.93	0.82	100
1 Been a very nervous person	1.98		5.60	0.80	0.55	72.75	0.60	100
2 Feel down in the dumps	2.11		5.75	0.70	0.40	84.13	0.62	100
3 Felt calm and peaceful	2.02		4.36	1.34	3.67	20.65	0.65	100
4 Felt downhearted and blue	2.37		5.30	0.95	0.87	51.90	0.68	100
5 Been a happy person	1.74		4.07	1.19	2.24	9.64	0.61	100
Health Transition	0.46	99.54	51.26	16.53	1.62	3.91	-	-
1 Change in health from one year ago	0.46		3.05	0.66	-	-	-	-
Physical Component Summary	4.52	95.48	49.49	10.16	-	-	-	-
Mental Component Summary	4.52	95.48	52.19	9.08	-	-	-	-

^a^RAND mean (SD) scores for bodily pain 76.90 (24.94) and general health perception 73.84 (21.45)

For the physical functioning scale, the easiest and most difficult items had the highest and lowest means respectively (Table 2). Item means increased with Guttman scale ordering across the two sets of items relating to climbing stairs and walking. The items “accomplished less than you would like” had the lowest means for the two role functioning scales. For vitality, the item “have a lot of energy” had the lowest mean score. For mental health the two items assessing positive affect had the lowest mean scores. The mental health item assessing the worst mental health state “so down in the dumps that nothing could cheer you up” had the highest mean score. The item score standard deviations were roughly equivalent within scales with the exceptions of the easiest and most difficult physical functioning items and the vitality and mental health scale items relating to positive and negative aspects of health.

The item-total correlations all exceeded the 0.4 criterion and in general were fairly similar in size with two exceptions including the easiest and most difficult physical functioning items. The two general health items relating to “I seem to get sick easier than others” and “I expect my health to get worse” also had somewhat lower correlations than the other items for this scale. With the exceptions of the physical functioning item relating to vigorous activities which had two correlations indicative of probable scaling success (within two standard errors) with the role-physical and general health scale items, there was 100% scaling success for all of the items. Cronbach’s alpha exceeded 0.8 for all scales and the physical functioning, role-physical and pain scales met the criterion for individual level analysis.

Less than 5% of respondents scored at the floor for six scales. The highest floor effect of 14.6% was for the role-physical scale, which together with the role-emotional scale also had the highest ceiling effects of 66.3 and 76.8% respectively. Ceiling effects were also high for the social functioning scale and over 35% for the physical functioning and bodily pain scales.

PCS and MCS were computable for 95.5% respondents with mean scores of 49.5 (10.2) and 51.2 (9.1) for the standard scoring.

### Normative data

Tables 3 and 4 give the normative data by gender for the different age groups. Table 3 is based on the standard scoring for the PCS and MCS [8] and Table 4 is based on the oblique (correlated) factor solution [26] and also includes the RAND scoring for bodily pain and general health. Across the age groups, females had lower scores than males, the only exceptions being small differences for physical functioning for 15–19 years, bodily pain for 20–29 years and general health for those over 79 years. Most of the differences were within two scale points up to the age group 50–59 years. However, females had lower scores of up to seven scale points for role-emotional in the age range 15–19 years. Much smaller differences were found for the remaining groups up to 50–59 years, where females scored two or more points lower for all scales with the exception above. For this and the older groups, the differences between the two genders generally increased for physical function, role-physical, bodily pain and social function with the largest differences for the oldest age group being for physical functioning at over 14 points. The difference for the remaining scales decreased for the two oldest age groups. The two youngest age groups had the highest scores for physical aspects of health; physical function, role-physical, bodily pain and general health. The age groups 40–49 and 60–69 years had the highest scores for role-emotional and mental health respectively.

**Table 3:** Mean SF-36 scale and summary scores based on standard scoring [8] by gender and age groups (*n* = 5396)

Age group	Sex		Physical function	Role-physical	Bodily pain	General health	Vitality	Social function	Role-emotional	Mental health	Physical summary	Mental summary
15–19	Male	*N*	*152*	*152*	*152*	*151*	*152*	*152*	*151*	*152*	*150*	*150*
		*Mean*	92.93	90.79	82.76	80.75	59.11	87.91	89.62	78.51	53.76	50.84
		*SD*	16.14	22.23	19.94	18.22	19.78	19.05	25.59	14.86	6.32	8.62
	Female	*N*	*142*	*143*	*144*	*144*	*144*	*144*	*144*	*144*	*141*	*141*
		*Mean*	93.30	88.23	79.34	77.74	52.95	84.81	81.71	74.23	53.49	47.69
		*SD*	14.21	24.47	20.82	19.26	18.18	19.62	31.00	15.07	7.10	9.62
	Total	*N*	*294*	*295*	*296*	*295*	*296*	*296*	*295*	*296*	*291*	*291*
		*Mean*	93.11	89.55	81.09	79.28	56.11	86.40	85.76	76.43	53.63	49.31
		*SD*	15.21	23.34	20.41	18.76	19.24	19.36	28.58	15.09	6.70	9.24
20–29	Male	*N*	*325*	*325*	*327*	*323*	*327*	*327*	*325*	*327*	*321*	*321*
		*Mean*	95.90	88.31	80.88	81.31	61.34	89.49	88.21	79.08	53.76	51.07
		*SD*	10.41	27.53	22.68	18.29	18.58	18.68	26.86	14.68	7.08	8.75
	Female	*N*	*399*	*399*	*400*	*397*	*398*	*401*	*399*	*398*	*395*	*395*
		*Mean*	94.40	87.93	81.03	79.93	58.27	88.93	87.55	78.75	53.41	50.67
		*SD*	11.44	27.28	22.07	17.78	18.02	17.26	27.22	13.85	7.07	8.87
	Total	*N*	*724*	*724*	*727*	*720*	*725*	*728*	*724*	*725*	*716*	*716*
		*Mean*	95.07	88.10	80.97	80.54	59.65	89.18	87.85	78.90	53.57	50.85
		*SD*	11.01	27.38	22.33	18.01	18.32	17.90	27.04	14.22	7.07	8.81
30–39	Male	*N*	*482*	*482*	*482*	*479*	*481*	*482*	*482*	*481*	*478*	*478*
		*Mean*	94.64	87.86	80.01	80.57	62.78	89.70	90.32	80.31	53.01	52.07
		*SD*	10.71	27.65	22.88	18.22	19.38	17.88	25.58	14.01	7.46	8.49
	Female	*N*	*533*	*533*	*534*	*530*	*534*	*534*	*530*	*534*	*525*	*525*
		*Mean*	92.47	82.83	77.28	79.82	58.36	87.29	86.98	79.00	52.04	51.02
		*SD*	13.67	33.24	24.26	20.66	20.24	20.49	28.77	15.46	8.90	9.56
	Total	*N*	*1015*	*1015*	*1016*	*1009*	*1015*	*1016*	*1012*	*1015*	*1003*	*1003*
		*Mean*	93.50	85.22	78.57	80.17	60.45	88.44	88.57	79.62	52.50	51.52
		*SD*	12.40	30.80	23.64	19.54	19.95	19.33	27.34	14.80	8.25	9.08
40–49	Male	*N*	*546*	*547*	*548*	*543*	*547*	*549*	*543*	*545*	*536*	*536*
		*Mean*	91.74	84.23	76.58	79.11	64.29	88.41	90.36	81.12	51.53	52.96
		*SD*	14.62	31.05	25.03	19.72	19.37	19.48	25.27	15.63	8.29	8.53
	Female	*N*	*530*	*529*	*529*	*525*	*529*	*531*	*526*	*529*	*518*	*518*
		*Mean*	89.93	80.99	72.38	77.23	59.76	87.03	87.67	80.20	50.10	52.15
		*SD*	16.11	34.42	25.60	21.65	20.65	20.74	28.41	15.56	9.81	9.56
	Total	*N*	*1076*	*1076*	*1077*	*1068*	*1076*	*1080*	*1069*	*1074*	*1054*	*1054*
		*Mean*	90.85	82.64	74.52	78.19	62.06	87.73	89.04	80.67	50.82	52.56
		*SD*	15.39	32.77	25.39	20.70	20.13	20.11	26.88	15.60	9.10	9.06
50–59	Male	*N*	*484*	*483*	*488*	*481*	*486*	*489*	*486*	*485*	*472*	*472*
		*Mean*	87.04	79.30	73.53	74.22	65.25	88.42	86.76	82.87	49.21	53.90
		*SD*	18.64	36.13	25.92	22.01	19.87	18.76	29.42	14.56	9.80	8.15
	Female	*N*	*490*	*488*	*489*	*486*	*487*	*490*	*484*	*488*	*475*	*475*
		*Mean*	82.38	71.11	66.36	70.81	58.30	83.06	84.33	80.44	46.40	52.59
		*SD*	21.21	40.47	28.06	24.27	22.21	24.28	32.86	16.22	11.40	9.18
	Total	*N*	*974*	*971*	*977*	*967*	*973*	*979*	*970*	*973*	*947*	*947*
		*Mean*	84.69	75.18	69.94	72.51	61.77	85.74	85.55	81.65	47.80	53.25
		*SD*	20.10	38.57	27.24	23.23	21.35	21.85	31.19	15.46	10.72	8.70
60–69	Male	*N*	*335*	*336*	*340*	*328*	*334*	*339*	*329*	*333*	*316*	*316*
		*Mean*	83.10	70.66	70.63	71.33	66.77	88.09	86.02	83.63	46.71	54.96
		*SD*	19.29	39.88	24.82	21.56	19.97	18.59	29.80	14.65	10.18	8.04
	Female	*N*	*334*	*331*	*332*	*321*	*329*	*333*	*329*	*329*	*313*	*313*
		*Mean*	75.42	62.41	65.08	67.26	59.81	84.87	77.51	80.42	43.93	53.08
		*SD*	23.16	42.41	26.69	23.64	21.72	21.90	35.41	16.04	11.86	9.38
	Total	*N*	*669*	*667*	*672*	*649*	*663*	*672*	*658*	*662*	*629*	*629*
		*Mean*	79.26	66.57	67.89	69.32	63.32	86.50	81.76	82.03	46.32	54.03
		*SD*	21.64	41.33	25.89	22.68	21.13	20.35	32.98	15.43	11.13	8.78
70–79	Male	*N*	*202*	*201*	*210*	*193*	*198*	*210*	*195*	*196*	*180*	*180*
		*Mean*	74.18	57.79	70.52	65.74	61.90	82.50	74.27	82.75	44.24	53.58
		*SD*	24.28	42.92	25.50	22.28	23.57	25.10	37.16	16.70	10.17	8.87
	Female	*N*	*224*	*226*	*228*	*208*	*219*	*227*	*219*	*214*	*195*	*195*
		*Mean*	63.28	46.61	61.84	63.29	56.69	77.09	60.27	78.53	40.84	51.10
		*SD*	27.20	43.94	29.73	22.89	22.80	26.05	44.32	17.96	11.35	10.12
	Total	*N*	*426*	*427*	*438*	*401*	*417*	*437*	*414*	*410*	*375*	*375*
		*Mean*	68.45	51.87	66.00	64.47	59.16	79.69	66.87	80.55	42.47	52.29
		*SD*	26.40	43.77	28.09	22.60	23.29	25.71	41.65	17.48	10.92	9.61
80+	Male	*N*	*69*	*69*	*72*	*65*	*69*	*74*	*71*	*67*	*61*	*61*
		*Mean*	60.16	34.54	65.39	59.80	55.68	75.84	55.40	79.68	38.31	50.83
		*SD*	27.31	41.35	25.30	23.91	22.76	28.39	42.89	17.61	10.47	10.13
	Female	*N*	*102*	*106*	*107*	*86*	*96*	*105*	*98*	*89*	*76*	*76*
		*Mean*	45.84	23.35	53.50	60.34	52.36	68.21	53.40	77.59	35.99	51.84
		*SD*	28.64	35.57	29.11	20.83	22.93	29.67	44.32	18.21	10.59	9.64
	Total	*N*	*171*	*175*	*179*	*151*	*165*	*179*	*169*	*156*	*137*	*137*
		*Mean*	51.62	27.76	58.28	60.11	53.75	71.37	54.24	78.49	37.02	51.39
		*SD*	28.90	38.23	28.18	22.13	22.85	29.31	43.61	17.93	10.56	9.83

**Table 4:** Mean SF-36 scale and summary scores based on alternative scoring [26] by gender and age groups (*n* = 5396)

Age group	Sex		Bodily pain	General health	Physical summary	Mental summary
15–19	Male	*N*	*152*	*151*	*150*	*150*
		*Mean*	86.07	79.39	53.36	49.77
		*SD*	18.91	18.47	6.34	8.95
	Female	*N*	*144*	*144*	*141*	*141*
		*Mean*	83.21	76.12	52.14	46.82
		*SD*	19.38	19.23	6.44	9.04
	Total	*N*	*296*	*295*	*291*	*291*
		*Mean*	84.68	77.79	52.77	48.34
		*SD*	19.16	18.88	6.41	9.10
20–29	Male	*N*	*327*	*323*	*321*	*321*
		*Mean*	83.86	79.81	53.31	50.08
		*SD*	21.08	18.44	6.49	8.78
	Female	*N*	*400*	*397*	*395*	*395*
		*Mean*	84.24	78.30	52.92	49.69
		*SD*	20.62	17.83	6.21	8.34
	Total	*N*	*727*	*720*	*716*	*716*
		*Mean*	84.07	78.98	53.10	49.87
		*SD*	20.81	18.11	6.34	8.53
30–39	Male	*N*	*482*	*479*	*478*	*478*
		*Mean*	83.04	79.10	53.04	50.74
		*SD*	21.29	18.21	6.90	8.56
	Female	*N*	*534*	*530*	*525*	*525*
		*Mean*	80.52	78.29	51.82	49.63
		*SD*	23.22	20.54	8.15	9.43
	Total	*N*	*1016*	*1009*	*1003*	*1003*
		*Mean*	81.72	78.68	52.40	50.16
		*SD*	22.35	19.46	7.60	9.04
40–49	Male	*N*	*548*	*543*	*536*	*536*
		*Mean*	79.73	77.62	52.05	51.14
		*SD*	23.89	19.57	7.90	9.37
	Female	*N*	*529*	*525*	*518*	*518*
		*Mean*	75.93	75.73	50.64	50.10
		*SD*	24.61	21.44	8.87	9.61
	Total	*N*	*1077*	*1068*	*1054*	*1054*
		*Mean*	77.86	76.69	51.36	50.63
		*SD*	24.31	20.52	8.41	9.50
50–59	Male	*N*	*488*	*481*	*472*	*472*
		*Mean*	76.79	72.84	50.36	51.60
		*SD*	24.87	21.69	9.32	9.28
	Female	*N*	*489*	*486*	*475*	*475*
		*Mean*	69.64	69.47	47.58	49.46
		*SD*	27.78	23.81	10.85	10.15
	Total	*N*	*977*	*967*	*947*	*947*
		*Mean*	73.21	71.15	49.97	50.53
		*SD*	26.60	22.83	10.21	9.78
60–69	Male	*N*	*340*	*328*	*316*	*316*
		*Mean*	74.21	70.00	48.50	51.73
		*SD*	23.90	21.15	9.41	9.03
	Female	*N*	*332*	*321*	*313*	*313*
		*Mean*	68.73	66.00	45.51	49.21
		*SD*	26.25	23.06	10.82	10.02
	Total	*N*	*672*	*649*	*629*	*629*
		*Mean*	71.50	68.02	47.01	50.48
		*SD*	25.22	22.19	10.24	9.61
70–79	Male	*N*	*210*	*193*	*180*	*180*
		*Mean*	73.94	64.57	45.60	50.32
		*SD*	24.74	21.74	10.50	10.50
	Female	*N*	*228*	*208*	*195*	*195*
		*Mean*	64.85	62.25	41.77	47.07
		*SD*	29.58	22.28	11.67	11.15
	Total	*N*	*438*	*401*	*375*	*375*
		*Mean*	69.21	63.37	43.61	48.63
		*SD*	27.71	22.02	11.27	10.95
80+	Male	*N*	*72*	*65*	*61*	*61*
		*Mean*	68.54	59.01	39.08	46.65
		*SD*	25.20	23.42	10.86	11.34
	Female	*N*	*107*	*86*	*76*	*76*
		*Mean*	56.12	59.25	37.59	45.76
		*SD*	29.61	20.06	10.23	10.95
	Total	*N*	*179*	*151*	*137*	*137*
		*Mean*	61.12	59.14	38.25	46.15
		*SD*	28.51	21.49	10.50	11.09

Across the age groups females had lower PCS and MCS scores, the only exception being for MCS in the age group over 79 years with the standard scoring (Table 3). The younger age groups had the highest PCS scores, which declined with successive age groups. For the standard scoring the MCS scores increased with successive age groups until the age group 60–69 and declined in the two older age groups. For the alternative scoring, MCS scores were very similar across the age groups above 15–19 years and there was a slightly sharper decline in scores for two oldest age groups compared to that for the standard scoring (5 versus 2.6 points).

## Discussion

This study was based on a general population survey from 2002–2003 [22] and provides more recent normative data for the Norwegian SF-36 version 1.2. This version of the SF-36 continues to be by far the most widely used in Norway together with normative data from 1996. The composition of the Norwegian general population has changed within this time, and the way individuals interpret and respond to items within health surveys also may have changed. Three Norwegian studies have used this more recent general population data for normative comparisons [23–25]. The current study is the first to assess this data for necessary measurement properties that have been widely applied in studies relating to normative data for the SF-36 including the IQOLA project [3].

The results of these analyses are an important prerequisite to publishing new normative data and using it for score interpretation. They show that the SF-36 has data completeness and that the instrument meets the criteria underlying the construction and scoring of multi-item scales [3]. Levels of missing data were low and scaling assumptions were met in this population. With the exception of one item relating to bodily pain, items had lower levels of missing data than for those for the Norwegian general population data collected as part of the IQOLA project [12]. The Scandinavian countries taking part in the IQOLA project had consistently higher levels of missing data across the 36-items than the other eight countries [3]. The present study found rates of missing data that were more in line with those for the other countries. All the correlations between the items and hypothesized scales met the criterion of 0.4. The levels of correlation were roughly equivalent with the same exceptions as those found in the IQOLA project [3]. Cronbach’s alpha was greater than the criterion of 0.7 for group analyses and met the criterion of 0.9 for individual analyses for three scales. The levels were comparable to those found for Norway in the IQOLA Project with a slightly higher range of 0.81–0.92 compared to 0.79–0.90 [3]. Item means within the scales were generally similar to the original Norwegian normative data [3]. Compared to the earlier norms, item means were slightly lower for physical functioning, role-physical, general health and role-emotional scales. They were slightly higher for vitality and mental health. The levels of floor and ceiling effects were broadly comparable to those found in the IQOLA project.

There are three possible reasons for the differences with the original Norwegian normative data. First, changes in the composition of the general population in the intervening period including age composition and an increased number of immigrants. Second, changes in the way in which individuals respond to SF-36 items which might follow increasing education and welfare levels. Third, this is the same version of the SF-36 as that used in the IQOLA project but subtle differences in the design and layout may have influenced responses. The former used an early standard layout for the SF-36 whereas the present survey used a slightly different more compact layout. It is only possible to speculate about the role of these different factors but together they represent good grounds for collecting and making available up-to-date normative data for widely used generic instruments including the SF-36.

Compared to the original normative Norwegian SF-36 data [12] this study has three important strengths. First, there are 3,000 more respondents in the current study compared to the original normative data, which makes the data a more suitable basis for interpreting SF-36 scores and changes in those scores for respondents with different health problems. Normative data has often a lower proportion of older respondents and particularly those aged 70 and over. Life expectancy continues to increase and an increasing proportion of applications of the SF-36 will include older people. The present study included 619 respondents in this age range who completed at least one SF-36 scale compared to just 227 for the original Norwegian normative data [12]. Moreover, there were 181 respondents aged over 79 years in the current study, which will improve the interpretation of SF-36 scores for older people with health problems. Second, during the two decades up to 2010, Norway has experienced better living standards coupled with changes in the composition of the general population including increasing numbers of immigrants, older people and increasing numbers of people living alone. Such changes will contribute to changes in the health status of the general population and therefore there is a need for more recent normative data. Third, the standard scoring for the SF-36 summary scales has been criticized [5, 26–28]. The current study includes normative data for both the PCS and MCS summary scores and the alternative RAND scoring for both these and the scales of bodily pain and general health. This normative data has not previously been reported for Norway. The alternative scoring algorithm is based on a correlated (oblique) physical and mental health factor model that is considered more appropriate given the moderate level of correlation found between physical and mental health [5, 26–28]. The authors of the alternative scoring algorithm recommend that weights be derived from other samples [26], which might include Norwegian data together with a comparison of weights based on the standard scoring. However, the use of the published US weights, as in the present study, enables comparisons with existing studies.

There are several possible study limitations. The main weaknesses of the present study are that it was not specifically designed for collecting normative data and the age of the data. Studies that are designed to collect normative data are costly and rarely undertaken. The study was pragmatic in its use of the most recent general population data available in Norway with a sufficient sample size. This data was used for comparative purposes in three recent Norwegian studies [23–25] which may be seen as a response to the need for more up-to-date normative data. It was therefore necessary to assess data completeness and to test the assumptions underlying the eight multi-item scales which comprise the SF-36 in this general population. The survey was part of a larger survey [22], which included home or telephone interviews with respondents prior to the postal survey described here. It is possible that prior contact including interviews may have influenced the response rate or responses to the postal questionnaire but assessment of such bias was not possible given the study design.

## Conclusion

In conclusion, more recent data for the SF-36 version one from a large scale survey of the Norwegian general population met important criteria described in the IQOLA Project [3]. The study found adequate evidence to support the use of the data for normative comparisons in Norwegian studies. It is recommended that this data is used in clinical and health services research for normative comparisons until more up-to-date general population data that are derived from a survey specifically designed for this purpose are available for the SF-36 in Norway.
